# Influence of obesity on remodeling of lung tissue and organization of extracellular matrix after blunt thorax trauma

**DOI:** 10.1186/s12931-020-01502-0

**Published:** 2020-09-17

**Authors:** Pengfei Xu, Fabian Gärtner, Adrian Gihring, Congxing Liu, Timo Burster, Martin Wabitsch, Uwe Knippschild, Stephan Paschke

**Affiliations:** 1 Department of General and Visceral Surgery, Surgery Center; Ulm University Medical Center, Albert-Einstein-Allee 23, 89081 Ulm, Germany; 2 Department of Biology, School of Sciences and Humanities, Nazarbayev University, Kabanbay Batyr Ave., 53, Nur-Sultan, 010000 Republic of Kazakhstan; 3 Division of Pediatric Endocrinology and Diabetes, Ulm University Hospital for Pediatrics and Adolescent Medicine, Eythstraße 24, 89075 Ulm, Germany

**Keywords:** Obesity, High fat diet, Thorax trauma, Extracellular matrix (ECM), Collagen, MMP activity, TIMP production

## Abstract

**Background:**

Previously, it has been shown that obesity is a risk factor for recovery, regeneration, and tissue repair after blunt trauma and can affect the rate of muscle recovery and collagen deposition after trauma. To date, lung tissue regeneration and extracellular matrix regulation in obese mice after injury has not been investigated in detail yet.

**Methods:**

This study uses an established blunt thorax trauma model to analyze morphological changes and alterations on gene and protein level in lean or obese (diet-induced obesity for 16 ± 1 week) male C57BL/6 J mice at various time-points after trauma induction (1 h, 6 h, 24 h, 72 h and 192 h).

**Results:**

Morphological analysis after injury showed lung parenchyma damage at early time-points in both lean and obese mice. At later time-points a better regenerative capacity of lean mice was observed, since obese animals still exhibited alveoli collapse, wall thickness as well as remaining filled alveoli structures. Although lean mice showed significantly increased collagen and fibronectin gene levels, analysis of collagen deposition showed no difference based on colorimetric quantification of collagen and visual assessment of Sirius red staining. When investigating the organization of the ECM on gene level, a decreased response of obese mice after trauma regarding extracellular matrix composition and organization was detectable. Differences in the lung tissue between the diets regarding early responding MMPs (MMP8/9) and late responding MMPs (MMP2) could be observed on gene and protein level. Obese mice show differences in regulation of extracellular matrix components compared to normal weight mice, which results in a decreased total MMP activity in obese animals during the whole regeneration phase. Starting at 6 h post traumatic injury, lean mice show a 50% increase in total MMP activity compared to control animals, while MMP activity in obese mice drops to 50%.

**Conclusions:**

In conclusion, abnormal regulation of the levels of extracellular matrix genes in the lung may contribute to an aberrant regeneration after trauma induction with a delay of repair and pathological changes of the lung tissue in obese mice.

## Introduction

Obesity is a rising issue in the modern world that does not only threaten industrial countries but has developed to a worldwide problem. Obesity leads to several comorbidities and can increase the mortality of many acute or chronic diseases [1]. Besides commonly known co-occurring conditions like type 2 diabetes, obesity can also impact respiratory diseases such as asthma, chronic bronchitis, pulmonary embolism as well as aspiration pneumonia in an adverse manner [2]. In addition to the enhanced prevalence of pathophysiological conditions, obesity serves as a supplementary risk factor after thorax trauma [3]. Acute respiratory distress syndrome (ARDS) is a common outcome after such a traumatic thorax injury. Due to acute hypoxemic respiratory failure, ARDS is a lethal factor that can occur in intensive care unit patients after thorax injuries [4–7]. However, it needs to be emphasised that multiorgan failure is the most common reason for mortality in ARDS patients. Usually a healthy wound healing process after lung trauma can be divided into three phases.

The first phase is described as the lung tissue injury phase and includes the acquisition of the trauma indicated by an edema caused by disruption of epithelial and endothelial cells.

The next phase is termed inflammation phase characterized by the recruitment of inflammatory immune cells, among them neutrophils, monocytes, and T-cells to the site of inflammation or injury via a CC-chemokine-ligand-2 (CCL2) gradient. After recruitment, they start to secrete fibroblast-activating cytokines as well as other growth factors, like macrophage colony stimulating factor (M-CSF) and granulocytes colony stimulating factor (G-CSF). The activated immune cells – mainly consisting of neutrophils and macrophages – start to remove the cell debris that occurred due to the traumatic injury. Moreover, the fibroblast population consisting of resident as well as bone marrow derived cells start proliferation as well as differentiation into myofibroblasts, which are responsible for the build-up of the extracellular matrix (ECM) by secretion of ECM components like collagens, elastins and gelatins.

The third and final phase is characterized as the repair phase. It is defined by the absorption of the alveoli fluid. The tissue architecture is re-established by substituting epithelial and endothelial cells and a contraction of fibroblasts, which results in a reduced wound size. Inflammatory cells as well as myofibroblasts enter apoptosis, which terminates the deposition of collagen. This is accompanied by a digestion of collagen by several enzymes including matrix metalloproteinases (MMPs) and a clearance caused by phagocytic cells.

All phases are highly coordinated and deregulation at any phase or a persisting stimulus of lung damage can lead to pathological deposition of collagen, often resulting in the development of fibrosis [8, 9].

ECM of the lung tissue mainly consists of elastin and collagen fibers, glycoproteins, and proteoglycans, whereby collagen displays the highest abundancy. Additionally, the two protein families MMPs and the tissue inhibitors of metalloproteinases (TIMPs) are important regulators of ECM organization [10].

While collagen is the main ECM component, there exist over twenty types of molecules, among them the collagen subtypes type I and III, which mainly contribute to the architectural structure of the alveoli wall, and collagen subtype IV which is the major component of basement membranes of the lung [11].

The regular ECM production and turnover is mainly regulated and coordinated by fibroblasts. Besides the build-up of the ECM by simple production of collagens, various enzymes, among them MMPs, are involved in ECM organization [11–14]. MMPs are zinc dependent proteases and produced by fibroblasts, epithelial cells, and immune cells able to degrade various subtypes of collagen as well as other elements of the ECM. Dependent on their substrates, MMPs are divided into elastases (MMP7 and 12), gelatinases (MMP2 and 9) and collagenases (MMP1, 8 and 13). Additionally, a group of membrane type MMPs (MMP14, 15, 16 and 17) can be defined [15–19]. MMPs can be inhibited by tissue inhibitors of metalloproteinases (TIMPs) consisting of four subtypes in humans (TIMP 1–4) [20]. It has been reported that the ratio of MMPs and TIMPs plays a crucial role in the amount of ECM in the tissue. The level of ECM increases if the activity or the amount of TIMPs rises or the quantity or activity of MMPs decreases [21]. This balance can be influenced by several factors, amongst them obesity [22].

At present, the impact of obesity on ARDS is still controversially debated. It was reported by Dossett et al. that a reduced probability of ARDS occurred in severely obese patients in comparison to patients with a normal body mass index (BMI) [23]. In contrast, several studies reported an enhanced risk of developing ARDS for obese patients, and therefore challenging the concept by Dossett et al. [24, 25]. They reported a decreased mortality rate due to mild ARDS (previously ALI, nomenclature changed 2012, Berlin ARDS definition) in patients suffering from obesity [25–27], although a higher mortality due to ARDS was revealed [28].

Generally, obesity is defined as a state of chronic inflammation, which is characterized by the constant over-production and release of pro-inflammatory adipocytokines, like leptin, resistin, visfatin as well as tumor necrosis factor alpha (TNF-α) [29].

Up to now it is uncertain if and how obesity can affect the damage, regeneration processes, tissue remodelling, and ECM organization after a blunt thorax trauma (BTT). Therefore, the main aim of this study is to investigate the differences of morphological changes of lung tissue as well as ECM build-up and its organization between normal weight (normal diet, ND) and obese (high fat diet, HFD) mice after lung injury by using a BTT mouse model. Gene analysis as well as evaluation on the protein level showed a deregulation of several ECM organizing proteins such as MMPs and TIMPs in obese mice post-BTT. HFD mice seem to lack a trauma related upregulation of ECM build-up and several ECM organizing proteins, which already starts at the gene level, but also can be translated to the protein and activity level. Our results provide evidence that abnormal regulation of extracellular matrix genes in the lung seem to contribute to a reduced MMP activity and a delayed repair of lung tissue in obese mice compared to normal weight mice.

## Material and methods

### Study design: animal model and breeding

All animal experiments were approved by the local and state authorities, license number 1183, Ulm University, and were carried out in accordance with local regulations and ARRIVE guidelines. As part of the animal application a power analysis was performed calculating the sample size for these experiments (nominal power: 0.8, nominal alpha: 0.025).

The presented work only focuses on male mice, since previous work with this mouse model revealed that a gender specific effect was not detectable regarding experiments performed for this study. Male C57BL/6 J mice received either a normal diet (ND, 10% kcal fat, DI12450, Research Diets Inc., by their European distributor Brogaarden® in Gentofte, Denmark) or a high fat diet (HFD, 60% kcal fat, DI12492, Research Diets Inc., by their European distributor Brogaarden® in Gentofte, Denmark) to generate normal weight or obese mice, respectively.

One week prior to breeding, parental mice received a ND or HFD to increase the susceptibility for obesity in the case of HFD [30]. Litters were weaned after three weeks, raised in the in-house breeding facility (Animal Research Center, Ulm University), and kept in a pathogen free facility before BTT induction, and in an individually ventilated cage (IVC) facility post-BTT. Mice were fed with the respective parental diet (ND or HFD) until the experiment was performed after 16 ± 1 week. The rearing was kept in a 12 h light/dark cycle at 22.5 ± 1 °C with ad libitum access to nutrition and water. For experiments, 16 ± 1 week old male mice were randomly, but diet-dependently grouped into control and blunt thorax trauma (BTT) mice.

### Study design: induction of thorax trauma

Prior to the injury, anesthesia was carried out in an anesthesia tube with a mixture consisting of 2.5 vol% sevoflurane (SevoraneTM Abbott, Germany) and 97.5% oxygen as well as analgesic buprenorphine (Temgesic®160 Reckitt Benckiser, Great Britain) subcutaneously injected with a syringe in a final concentration of 0.03 mg/kg. Mice were fixed on an acrylic glass plate in a supine position and the chest was shaved. At this point the experiment ended for the control mice, while the trauma mice received a BTT induced by a modified mice single blast wave system described by Knöferl et al. [31]. The blast wave generator was directed with the nozzle towards the mouse’s chest (1.5 cm distance) releasing a reproducible single blast wave causing the trauma. Control mice were treated equally but did not obtain BTT.

Mice received their regular diet until they were sacrificed with CO_2_ 1 h, 6 h, 24 h, 72 h and 192 h post-BTT. The lung was isolated and stored at − 80 °C after rapid freezing in liquid nitrogen or fixed in 4% formaldehyde and embedded in paraffin (Thermo Fisher Scientific, Germany).

### Hematoxylin and eosin (HE) staining

Formaldehyde fixed lung tissue from male ND and HFD mice was collected from the control group as well as 1 h, 6 h, 24 h, 72 h and 192 h post-BTT and subsequently embedded in paraffin (Sakura, Germany). 5 μm thick tissue-sections were deparaffinized, rehydrated in decreasing alcohol series and subsequently stained in hematoxylin for three minutes. Afterwards the sections were blued using running water for 10 min and subsequently stained in eosin for 10 s [32]. The staining was completed by implementing dehydration of the slides in increasing alcohol series with incubation in Roti®-Histol. Sections were mounted with Entellan®. One area per animal out of a total of 6 animals per group was analyzed for the evaluation of the trauma. This number is based on the power analysis conducted for the animal experiment application (license number: 1183). Pictures were taken with the UC30 color camera at X10 and X40 magnification. All stainings were acquired with an Olympus IX81 microscope and analyzed with the Olympus software cellSens Dimensions 2.3 (Build 18,987).

### Lung injury score

Lung injury score was determined to evaluate the impact of the BTT in lean and obese mice. The calculation was performed based on the HE staining of BTT mice 6 h post trauma. The used formula for calculation shown below is based on the lung injury score published by *Malute-Bello* et al [33].

$$ \mathrm{Injury}\ \mathrm{score}=\frac{\left[\left(\frac{\mathrm{alveolar}\ \mathrm{hemorrhage}\ \mathrm{points}}{\mathrm{no}.\kern0.5em \mathrm{of}\ \mathrm{fields}}\right)+2\ast \left(\frac{\mathrm{alveolar}\ \mathrm{infiltrate}\ \mathrm{points}}{\mathrm{no}.\mathrm{of}\ \mathrm{fields}}\right)+3\ast \left(\frac{\mathrm{fibrin}\ \mathrm{points}}{\mathrm{no}.\kern0.5em \mathrm{of}\ \mathrm{fields}}\right)+\left(\frac{\mathrm{alveolar}\ \mathrm{septal}\ \mathrm{congestion}}{\mathrm{no}.\kern0.5em \mathrm{of}\ \mathrm{fields}}\right)\right]}{\mathrm{total}\ \mathrm{number}\ \mathrm{of}\ \mathrm{alveoli}\ } $$

The definitions of each score in association which its specific category is depicted in Table 1.

**Table 1:** Quantitative evaluation of the lung injury after BTT based on alveolar hemorrhage (infiltration of red blood cells (rbcs)), alveolar infiltration (intra-alveolar cells), intra-alveolar fibrin and congestion of alveolar septae

Tissue	0	1	2	3
Alveolar hemorrhage	None	At least 5 rbcs/alveolus in 1–5 alveoli	At least 5 rbcs/alveolus in 5–10 alveoli	At least 5 rbcs/alveolus in > 10 alveoli
Alveolar infiltration	No infiltration	< 5 intra-alveolar cells/field	10–20 intra-alveolar cells/field	> 20 intra-alveolar cells/field
Intra-alveolar fibrin	None	Fibrin in < 1/3	Fibrin in 1/3–2/3	Fibrin in > 2/3
Alveolar septae	All thin and delicate	Congested in < 1/3	Congested in 1/3–2/3	Congested in > 2/3

### Sirius red (SR) staining

The deparaffinized lung sections were stained using SR solution (0.1% in aqueous saturated picric acid) for 1 h and differentiated in 0.5% acetic acid. Subsequently, the slides were counterstained with hematoxylin for 10 min to stain the cell nuclei as described by Xu et al. [32]. Increasing alcohol series and Roti-Histol® was used for dehydration of the sections that were then covered with Entellan®. One area per animal out of a total of 6 mice per group was analyzed for the evaluation of the trauma. This number is based on the power analysis conducted for the animal experiment application (license number: 1183). Pictures were taken with the UC30 color camera at X10 and X40 magnification. All stainings were acquired with an Olympus IX81 microscope and analyzed with the Olympus software cellSens Dimensions 2.3 (Build 18,987).

### Determination of collagen content

Determination of collagen content in lung tissue was performed with the Total Collagen Assay Kit (Abcam, Germany) according to manufacturer specifications. Frozen lung tissue sections were crushed and 100 μL distilled H_2_O per 10 mg tissue were added. Samples were homogenized using a tissue homogenizer and 100 μL of suspension were transferred in a pressure-tight, thermally stable, screw-capped tube. Standard preparation, alkaline hydrolysis, chemical reaction, and measurement were conducted according to the manufacturer’s instructions.

### Quantitative real-time polymerase chain reaction (qPCR)

RNA from frozen lung tissue was isolated using a RNeasy®Mini Kit according to the manufacturer’s specifications. 100 ng of total RNA was transcribed to cDNA by using the AffinityScript cDNA Synthesis Kit (Agilent Technologies, Santa Clara, USA). The generated cDNA was tested for genomic contamination by a ß-actin control PCR. A successful generation of cDNA was shown by using a primer pair targeting the exon sequence of *ß-actin* (forward primer: 5′-GGT ATC CTG ACC CTG AAG TA-3′, reverse primer: 5′-GTC AGG CAG CTC ATA GCT CT-3′). To exclude genomic contamination a primer pair targeting the intron sequences of *ß-actin* was used (forward primer: 5′-CGA GCA GGA GAT GGC CAC TGC-3′, reverse primer: 5′-GTG AGC TCT CTG GGT GCT GGG-3′). The uncontaminated cDNA was used for qPCR analysis using *Gapdh* (Mm_Gapdh_3_SG QuantiTect Primer Assay, QT01658692, Qiagen) as the reference gene [34]. Additionally, the following primer were used for gene analysis: *Fn1* (Mm_Fn1_1_SG QuantiTect Primer Assay, QT00135758, Qiagen), *Col1a* (Mm_Col1a1_1_SG QuantiTect Primer Assay, QT00162204, Qiagen), *Col3a* (Mm_Col3a1_1_SG QuantiTect Primer Assay, QT01055516, Qiagen), *Mmp*2 (Mm_Mmp2_1_SG QuantiTect Primer Assay, QT00116116, Qiagen), *Mmp*8 (Mm_Mmp8_1_SG QuantiTect Primer Assay, QT00113540, Qiagen), *Mmp*9 (Mm_Mmp9_1_SG QuantiTect Primer Assay, QT00108815, Qiagen), *Timp*1 (Mm_Timp1_1_SG QuantiTect Primer Assay, QT00996282, Qiagen) and *Timp2* (Mm_Timp2_1_SG QuantiTect Primer Assay, QT00138558). Reference gene, genes of interest and SYBR R Green (QantiTect R SYBR R Green PCR Kit) were obtained from Qiagen (Hilden, Germany). Gene expression was measured in double determination. Gene expression profiles of genes are calculated using the ΔΔCT method. Results are shown as fold change ± SEM. Since the ΔΔCT method sets the control mice to a value of 1, these values were excluded from the graph; however, a dotted line is used as an indication of the control level. Data was analyzed using the log of the fold change and a two-way ANOVA followed by an uncorrected Fisher’s LSD test (*n* = 6). The log was used for statistical analysis to avoid skewness of the data.

### Gelatin zymography

The lung samples of six ND and HFD male mice, including control mice as well as mice after 1 h, 6 h, 24 h, 72 h and 192 h of trauma induction were used for the gelatin zymography. 50 mg of tissue was dissolved in 0.5 ml ice-cold NP-40 lysis buffer (50 mM Tris-HCl, 120 mM NaCl, 1% NP-40, 10% glycerol) for the generation of the protein extract. Liquid N_2_ frozen parts of skeletal muscle tissue were crushed with a pestle and subsequently homogenized in lysis buffer on ice for 30 min. The homogenate was centrifuged in a microcentrifuge at 16.000 x g for 20 min at 4 °C, supernatant was collected, and the protein concentration was measured with a Pierce BCA Protein Assay Kit (23,225, ThermoFisher, Germany). 10 μg protein of each sample was used for electrophoresis and resolved in non-reducing sample buffer. Samples were loaded on a 7.5% acrylamide gel containing 5 mg/ml gelatin. After electrophoresis, the gel was incubated in renaturing buffer (2.5% Triton X-100, 50 mM Tris-HCl pH 7.5, 5 mM CaCl_2_, 1 μM ZnCl_2_) for 30 min at room temperature (RT). The gel was developed in developing buffer (1% Triton X-100, 50 mM Tris-HCl pH 7.5, 5 mM CaCl_2_, 1 μM ZnCl_2_) for 30 min at RT. Subsequently, a 16–18 h incubation with fresh developing buffer was performed at 37 °C. The gels were stained with Coomassie Blue staining solution, acquired with Vilber Fusion FX and evaluated with Image J. All samples were measured in triplicates.

### Determination of total MMP activity

Total MMP activity was determined using the MMP activity assay kit (ab112146, Abcam, Germany) according to the manufacturer’s specifications. 5 μg lung tissue lysate was tested from each timepoint from ND and HFD male mice and measured in duplicates. The tissue lysate was first activated with equal volumes of 2 mM APMA working solution and then mixed with MMP Green Substrate. An end point reading was performed after 30 min of incubation time. The fluorescence intensity was monitored at Ex/Em = 490/525 nm. Baseline was determined with a negative control and subtracted from the individual samples to receive the measured activity of the MMPs.

### IHC staining of TIMP1/2

Paraffin-embedded, 1 μm thick cross-sections of lung tissue were dried for 24 h at 40 °C. Sections were subsequently deparaffinized in Roti-Histol® (6640.4, Carl Roth). Hydration was performed in decreasing alcohol series before they have been used for IHC staining. For antigen demasking, sections were boiled in citrate demasking solution (14746, CST) in a microwave at 450 W for 2 min and subsequently at 270 W for 20 min. Sections were cooled and several washes with water as well as Tris-Triton buffer (0.1% Triton) were performed before applying a blocking step, which was performed using peroxidase blocking solution (S202386–2, Agilent). Sections were washed in PBS and subsequently stained overnight at 4 °C with primary antibodies either against TIMP1 (ITT4658, G-Biosciences, 1:500) or TIMP2 (ITA6535, G-Biosciences, 1:200). PBS was used for diluting the antibodies. The sections were washed, and the secondary staining was performed with two drops Histofine Simple Stain Max Po Anti-Rabbit (414142F, medac diagnostika). Sections were incubated for 30 min at RT. Subsequently, sections were washed and incubated with peroxidase substrate AEC (K3461, Dako). The samples were washed, counterstained with hemalaun (109,249, Sigma-Aldrich), blued with tap water and finally mounted with aqueous permanent mounting medium (S196430–2, Agilent). Three areas per animal out of a total of 6 mice per group were analyzed. Pictures were taken with the UC30 color camera at X10 magnification. All stainings were acquired with an Olympus IX81 microscope and analyzed with the Olympus software cellSens Dimensions 2.3 (Build 18,987).

### Statistical evaluation

Statistical analysis was carried out using Graphpad Prism (Prism 7.04, GraphPad Software, Inc., La Jolla, CA, USA). For each individual analysis, the used test is depicted below the graph. In general, a two-way analysis of variance (ANOVA) followed by an uncorrected Fisher’s LSD test (α = 0.05) was used if several time- or diet-dependent comparisons were performed. If only specific time-points were compared (weight and lung injury score 6 h after trauma) a one-tailed, unpaired t-test was performed (α = 0.05). Data is depicted as Mean ± SEM. The following indicators were used for all statistical tests: * indicates *p* < 0.05, ** indicates *p* < 0.01, *** indicates *p* < 0.001, **** indicates *p* < 0.0001.

## Results

**Fig. 1:**
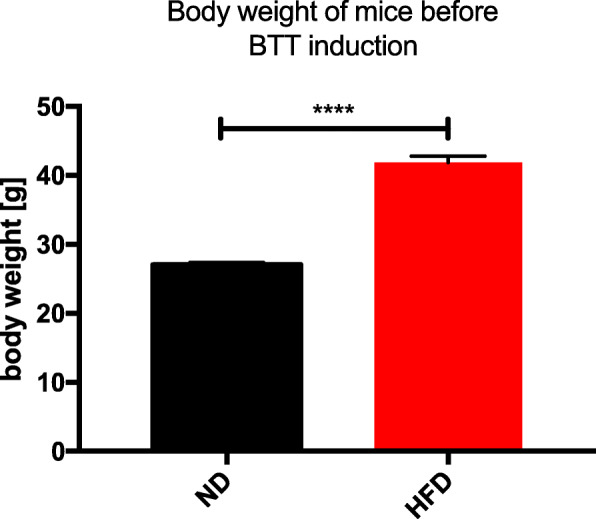
Body weight distribution of normal weight and obese mice. Values are given as mean ± standard error of the mean (SEM). Statistical analysis was done by two-way ANOVA, where **** indicates *p* ≤ 0.0001

A high-fat diet induced obesity mouse model was used to investigate the influence of obesity on lung regeneration after BTT focusing on post-traumatic morphological changes, collagen deposition and ECM organization. Weight differences between normal and diet-induced obese mice are shown in Fig. 1. BTT was induced in normal weight and obese, 16 ± 1-week old, male C57BL/6 J mice using a single blast wave system described by Knöferl et al. [31].

### Initial damage of lung tissue after BTT induction is comparable between ND and HFD animals

**Fig. 2:**
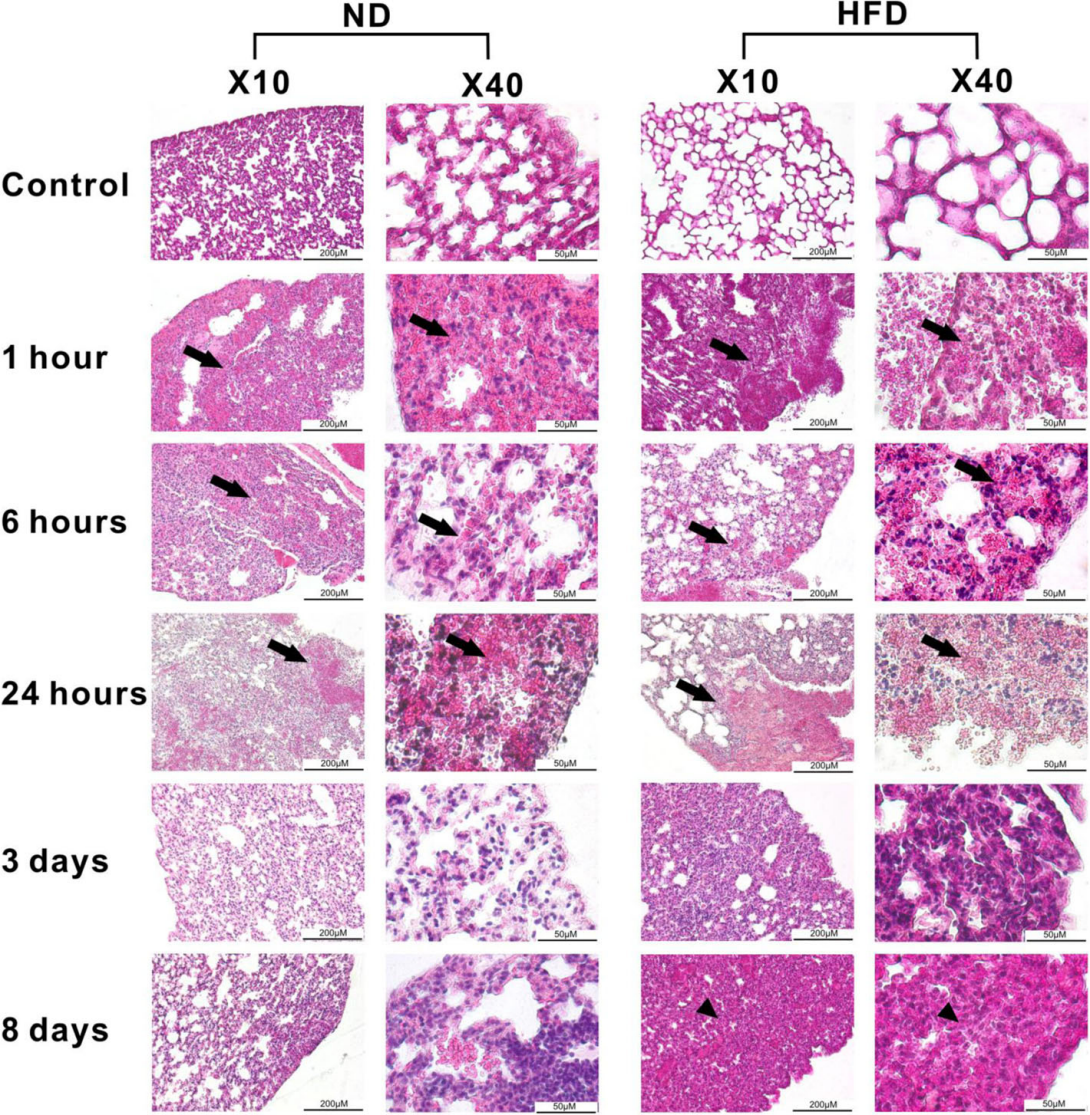
Regeneration process of the lung after blunt thorax trauma (BTT). Hematoxylin-eosin (HE) staining of lung tissue sections of male lean and obese C57BL/6 J mice after induction of blunt thorax trauma (*n* = 6). Figure shows lung tissue sections from control animals as well as lung sections from1 h, 6 h, 24 h, 72 h and 192 h post trauma. Arrow = lung tissue damage, arrowhead = lung tissue with intra-alveoli debris, thickened alveoli wall and alveoli collapse. Pictures were taken with an Olympus IX81microscope using Xcellence v.1.2. Scale = 50 μm at 40x magnification or 200 μm at 10x magnification. Abbreviations: HFD, high fat diet; ND, normal diet

Morphological evaluation of lung tissue injury and regeneration after BTT revealed that tissue morphology of ND and HFD control mice was similar showing clearly structured, distinct alveoli (Fig. 2). As a first, diet-independent response to injury, disruption of lung parenchyma was visible during the DI phase post-BTT induction. This was characterized and indicated by damaged alveoli with an irregular alveoli shape, fluid filled alveoli, and interstitial edema, as well as infiltrated erythrocytes (pink color) and immune cells (dark blue) (Fig. 2). This effect was observed in both, lean and obese mice. To further confirm that the initial tissue damage caused by the BTT is comparable between the two diets a lung injury score was used to quantitatively access the degree of damage. The lung injury score, which is based on the four factors alveolar hemorrhage, alveolar infiltration, intra-alveolar fibrin and alveolar septae (see Material & Methods), was calculated using HE-stained lung tissue sections from 6 h post trauma (*n* = 6, 10 areas per animal and at least 300 alveoli) and revealed that the initial damage is comparable between ND (Mean ± SEM, 8.276 ± 1.186) and HFD (Mean ± SEM, 9.45 ± 0.7256) mice.

However, differences during the R phase were observable. At later time-points post-BTT, tissue debris was mainly removed from alveoli of ND mice, whereas in HFD mice intra-alveoli debris, thickening of the alveoli wall and alveoli collapse of the lung persisted in the area of the trauma. Therefore, HFD mice seem to respond differently to the trauma compared to lean mice and based on the level of tissue regeneration seem to lack the proper response to trauma.

### Decreased gene response of ECM components in HFD mice post-BTT

**Fig. 3:**
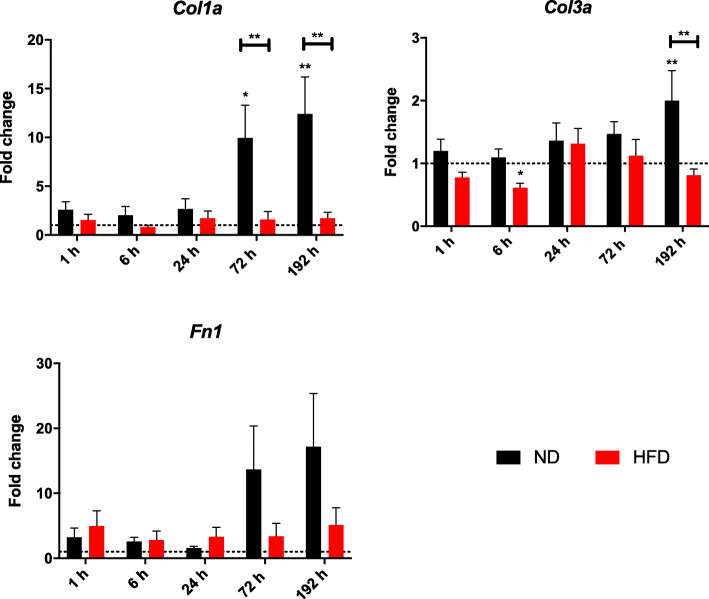
Gene expression status of lung tissue regarding ECM build-up post-BTT. Expression levels of collagen 1, collagen 3 and fibronectin in lung tissue were determined by qPCR using *Gapdh* as a housekeeping gene. *Col1a*, *Col3a* and *Fn1* show the gene expression differences when comparing all the time-points after lung injury to the respective controls. Statistical significance was determined using two-way ANOVA followed by an uncorrected Fisher’s LSD test (α = 0.05). * indicates *p* < 0.05, ** indicates *p* < 0.01, *** indicates *p* < 0.001, **** indicates *p* < 0.0001. Indicators of significance directly above the bars of the diagram indicate a statistical difference to the control, while stars above the connector line show differences between the two diets at that specific time point

Based on previous studies regarding the influence of diet-induced obesity on tissue regeneration in a blunt muscle trauma model [32], it is known that obese mice show a decreased response to the muscle trauma on gene level regarding ECM build-up components like collagens and fibronectin. Therefore, a quantitative gene expression analysis of ECM components including the genes of collagen 1a (*Col1a*)*,* collagen 3a (*Col3a*) and fibronectin (*Fn1*) was conducted (Fig. 3) to transpose these published findings from the muscle trauma to the lung trauma.

Gene expression analysis showed significantly increased levels of *Col1a* (Fig. 3) in ND mice during the R phase post-BTT compared to the respective controls. This upregulation was not present in HFD mice (Fig. 3) leading to a significant difference between the diets 72 h and 192 h post trauma.

*Col3a* shows a constant time-dependent increase of its gene expression level in ND mice leading to a statistically significant increase 192 h post-BTT. This increasing trend was not detectable in HFD mice (Fig. 3). After a significant decrease of *Col3a* 6 h after trauma induction, the gene level returns to baseline and remains unaffected by the influence of the trauma. The gene level of *Fn1* was comparable to *Col1a*. A significant increase in gene expression of *Fn1* was observed in ND mice at the R phase post-BTT compared to control mice. This change in gene expression was not detectable in HFD mice (Fig. 3).

Gene expression analysis of *Col1ɑ*, *Col3ɑ* and *Fn1* would hint at an increased expression of ECM components in ND mice post-BTT in the later stage of regeneration (R phase) in comparison to control mice. In contrast, the gene expression of these genes in HFD mice seem to respond to the trauma in a decreased manner.

### Differences in gene expression of ECM build-up genes do not influence the collagen deposition in the lung

**Fig. 4:**
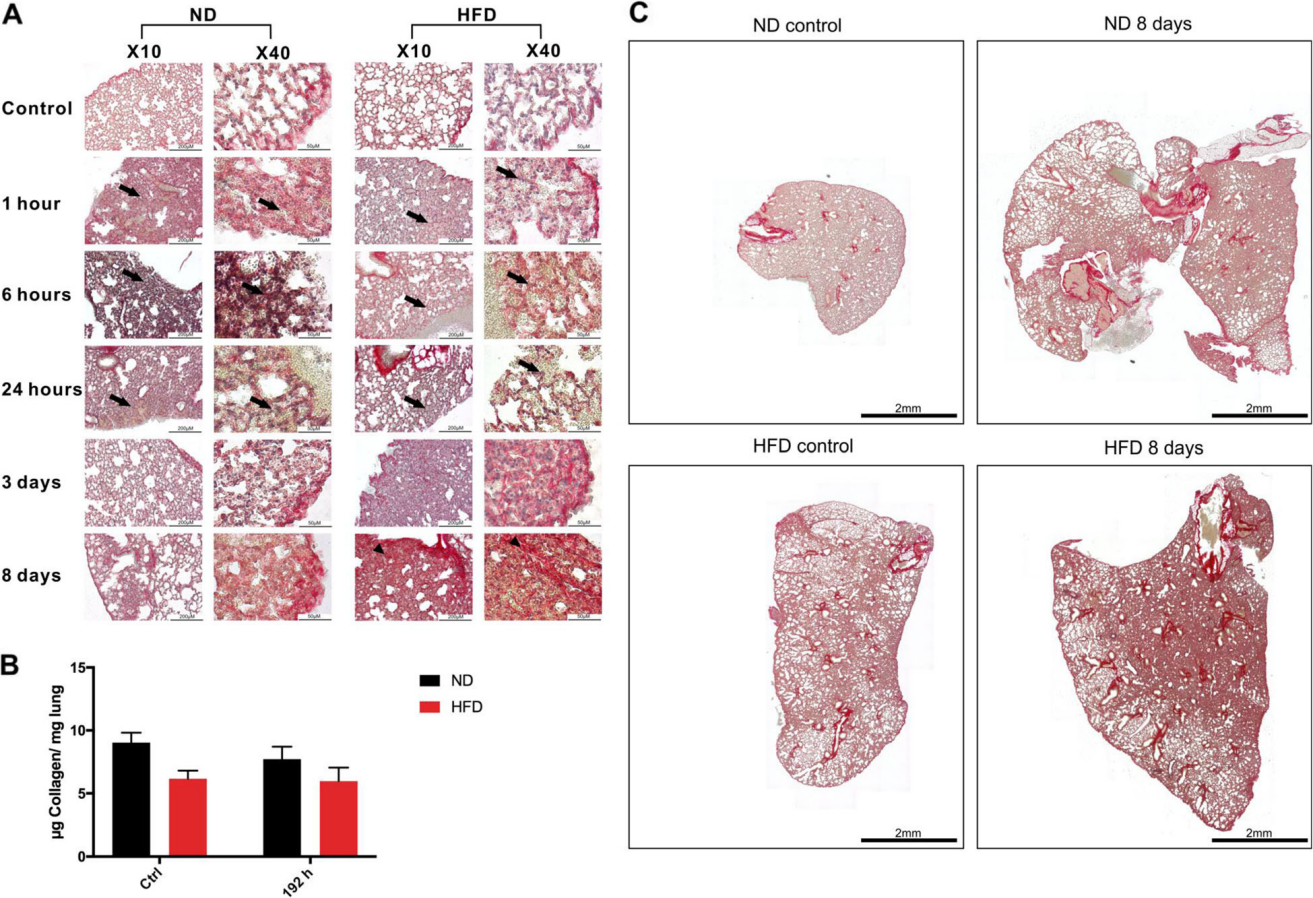
ECM remodeling in lung tissue after BTT. A) ECM regulation and collagen amount in lung tissue post-BTT in ND and HFD mice. Sirius Red staining of lung tissue sections. Arrowhead = fibrosis, arrow = damaged lung tissue with blood cell infiltration. Pictures were taken with an Olympus IX81 using Xcellence v.1.2. Scale = 50 μm at 40x magnification or 200 μm at 10x magnification. B) Quantification of collagen in the lung using a chemical collagen quantification assay kit. Results are presented as mean ± SEM. C) Schematic presentation of Sirius Red stained whole lung tissue sections. Abbreviations: HFD, high fat diet; ND, normal diet

Sirius Red (SR) staining was used to visually evaluate the deposition of collagen after BTT as part of building up the ECM and starting the regeneration of the damaged tissue (Fig. 4a). In addition to a visual evaluation of the SR stainings, the amount of collagen in lung tissue was colorimetrically quantified 192 h post-BTT (Fig. 4b). The SR stainings indicate an increase of collagen deposition in both, lean and obese mice as a response to the trauma (Fig. 4a). Based on visual assessment of SR sections on a local level (Fig. 4a) and a more comprehensive level (Fig. 4c), collagen deposition increased in the lung of ND and HFD mice leading to an expansion of collagen 192 h post-BTT. However, colorimetrical quantification of total collagen amount in the tissue could not confirm this observation (Fig. 4b). This can be explained by either local disposition of collagen, which cannot be detected due to the nature of the colorimetric analysis by measuring total amount in a bigger piece of the lung. Another explanation could be the normalization to the total weight of the analyzed lung tissue. If the amount of collagen increases, but due to the influx of the observed interstitial cells (Fig. 2), the density of the tissue increases as well, the colorimetric analysis would show no change. Nevertheless, neither visual assessment of the SR sections nor colorimetrical collagen quantification showed the same results as the gene expression analysis of the ECM build-up genes. The significant increase of the gene levels of *Fn1* and *Col1a/Col3a* in lean mice compared to HFD mice did not translate to the protein level, since no difference between the two diets can be observed based on collagen deposition.

### Decreased gene level of ECM organizing genes in the lung of HFD mice post-BTT

**Fig. 5:**
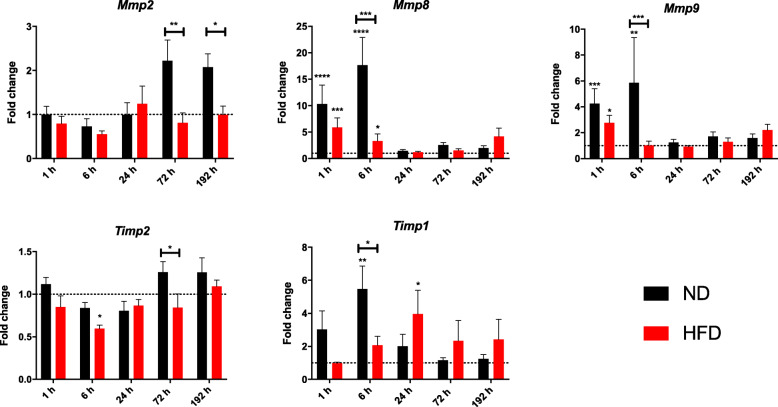
Gene expression status of Mmps and Timps in lung tissue post-BTT. Expression levels of *Mmp2*, *Mmp8*, *Mmp9*, *Timp2* and *Timp1* in lung tissue were determined by qPCR using *Gapdh* as a housekeeping gene. *Mmp2*, *Mmp8*, *Mmp9*, *Timp1*and *Timp2* show the gene expression differences by comparing all the time-points after lung injury to their respective controls. Statistical significance was determined using two-way ANOVA followed by an uncorrected Fisher’s LSD test (α = 0.05). * indicates *p* < 0.05, ** indicates *p* < 0.01, *** indicates *p* < 0.001, **** indicates *p* < 0.0001. Indicators of significance directly above the bars of the diagram indicate a statistical difference to the control, while stars above the connector line show differences between the two diets at that specific time point

Since the gene level of ECM build-up components in the lung showed a significant decrease in obese mice as a response to the trauma, but the outcome on protein level did not show any difference between the two diets, it hints towards a possible deregulation of ECM organizing genes. The group of MMPs and their tissue inhibitor of metalloproteinases (TIMPs) are such factors that regulate the build-up and break down of the ECM. Therefore, a quantitative gene expression analysis of genes involved in the organization and remodeling of ECM, including *Mmp2*, *Mmp8*, *Mmp9* and *Timp1*, and *Timp2* was implemented (Fig. 5).

These genes can be assorted to the previously mentioned DI and R phase. During the initial response of the DI phase at 1 h and 6 h post-BTT, gene expression levels of *Mmp8, Mmp9* as well as the respective inhibitor *Timp1* were upregulated in lung tissue of mice after trauma independently of the diet. However, several differences between the diets can be described. *Mmp8* is increased in ND mice 1 h and 6 h post-BTT, but HFD mice start to return to baseline already at 6 h post trauma, leading to a statistically significant difference between the two diets. 24 h post trauma both diets returned to baseline. *Mmp9* shows the same trend, being upregulated at early time-points in both diets and returning to baseline in HFD mice 6 h after trauma resulting in a statistically significant difference in the gene expression level of *Mmp9* between the diets. The gene level of the respective inhibitor, *Timp1*, shows an early response as well. However, the response seems to be shifted in obese mice. While the gene level is peaking 6 h post trauma in ND mice, HFD show the highest expression at 24 h. Additionally, the gene level of *Timp1* does not go back to baseline at later time points.

*Mmp2* and its inhibitor *Timp2* can be assorted to the R phase. *Mmp2* is a late responder gene and its expression is increased in ND mice at later timepoints namely 72 h and 192 h. This response seems to be missing in obese mice leading to a statistically significant difference between the two diets. *Timp2* did not show a noticeable response to the trauma, however a drop in gene expression can be noticed in HFD mice 6 h post trauma. This is accompanied by a drop in expression level of *Mmp2* as well resulting in the same ratio of *Mmp2/Timp2*.

HFD mice showed a diminished response to the trauma based on the gene expression of *Mmp2*, *Mmp9* and *Mmp8*. Additionally, obese mice showed a shifted and delayed response in the upregulation of the *Mmp8/9* inhibitor*, Timp1* (Fig. 5).

### Decreased activity of MMP9 in lung tissue of HFD mice after trauma

**Fig. 6:**
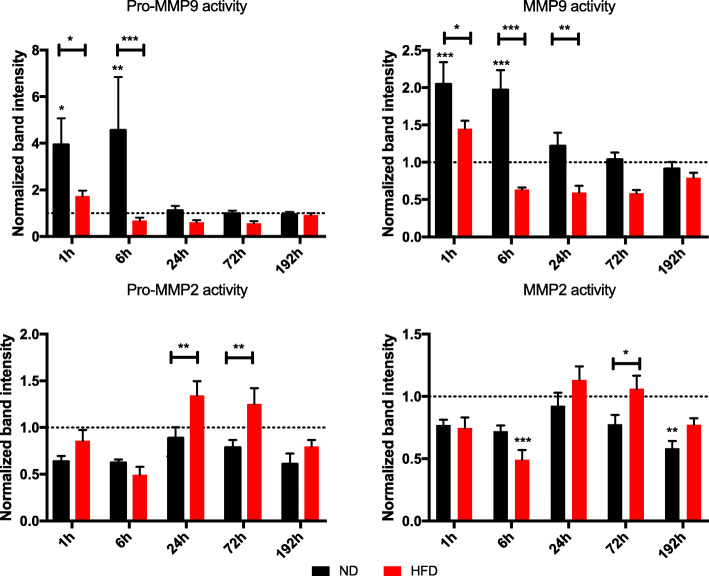
Activity of pro−/MMP9 and pro−/MMP2 in lung tissue of lean and obese mice after BTT. Activity of Pro-MMP9, MMP9, Pro-MMP2 and MMP2 was evaluated using gelatin zymography. Lung tissue from control animals as well as 1 h, 6 h, 24 h, 72 h and 192 h post trauma were used for this analysis. Statistical significance was determined using two-way ANOVA followed by an uncorrected Fisher’s LSD test (α = 0.05). * indicates p < 0.05, ** indicates p < 0.01, *** indicates p < 0.001, **** indicates p < 0.0001. Indicators of significance directly above the bars of the diagram indicate a statistical difference to the control, while stars above the connector line show differences between the two diets at that specific time point

The activity of MMP2 and MMP9 was determined using gelatin zymography to translate the findings regarding diminished responses on gene level of *Mmps* to protein level (Fig. 6). Both, MMP2 and MMP9, have precursor proteins, pro-MMP2 and pro-MMP9, which can be detected with this method as well. Although pro-MMPs are naturally inactive, SDS denatures the proteins during electrophoresis and leads to a dissociation of Cys^73^ from the zinc molecule, which leads to its activation [35] and the possibility of measuring its activity with a gelatin zymography.

Gelatin zymography confirmed the roles of MMP9 as an early responder and MMP2 as a late responder as it was proposed by the gene expression analysis. The level of pro-MMP9 follows in general the detected activity of MMP9, its activity is increased in the early time-points in both lean and obese mice. However, the activity returns to baseline in obese mice after 6 h, while it is still increased in lean mice leading to a statically significant difference between the diets. After 24 h the activity of pro-MMP9 and MMP9 is back to baseline in both diets. This confirms the gene expression analysis results indicating a decreased response to the trauma by obese animals regarding MMP9.

MMP2, which was a late responder during gene level analysis, did not show a clear response on protein level to the trauma. A drop of activity in obese mice 6 h after trauma which was already observed on gene level can be detected in this activity assay as well. However, no increase of activity during late timepoints can be detected in lean or obese mice. In contrast, during later timepoints (72 h and 192 h) the level of activity is higher in obese mice compared to lean animals, although this difference is statistically significant it is most certainly biological insignificant. The activity data of MMPs indicates a decreased remodeling response of obese animals, but this data would not be complete without elucidating the protein levels of the respective inhibitors TIMP1 and TIMP2.

### Decreased expression of TIMP2 in lung tissue of HFD mice during the R phase after-BTT

**Fig. 7:**
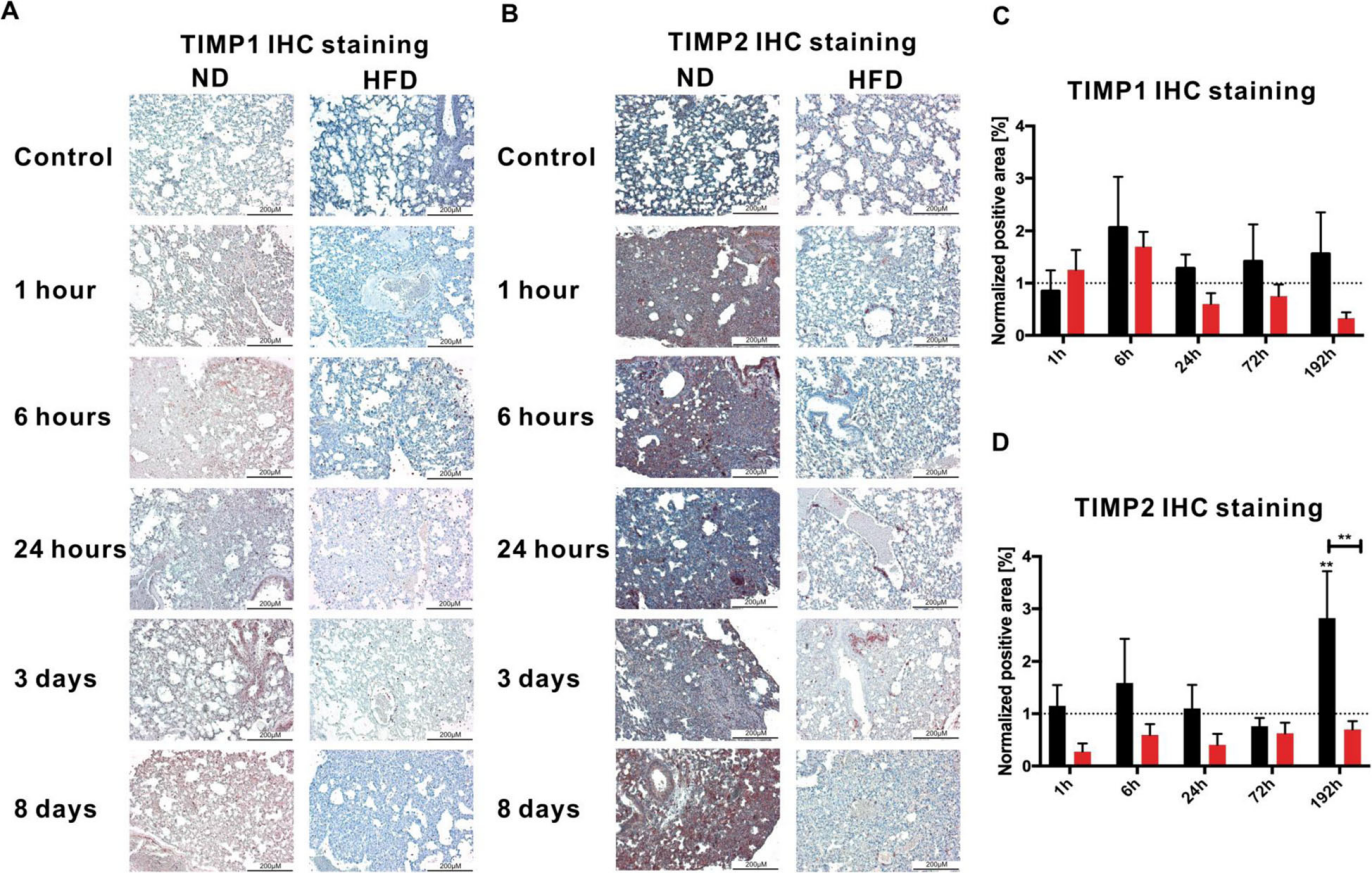
TIMP1/2 IHC staining of lung from lean and obese mice after BTT. A) TIMP1 and B) TIMP2 staining in lean and obese mice from control animals as well as 1 h, 6 h, 24 h, 72 h and 192 h post trauma. The area of positive staining was measured and is depicted as values next to the respective depiction of the staining for TIMP1 (C) and TIMP2 (D). The data is presented as mean ± SEM. Statistical significance was determined using two-way ANOVA followed by an uncorrected Fisher’s LSD test (α = 0.05) ** indicates p < 0.01

The translation of the gene data regarding *Timp1/2* to the protein level was achieved with IHC staining (Fig. 7a/b). TIMP1 staining (Fig. 7a) shows an increase of protein expression 6 h after BTT in both diets. Even though TIMP1 decreases under its baseline in obese animals after 24 h post trauma, the difference between the two diets is never statistically significant although the protein expression of TIMP1 in the lung of obese animals always stays below the level of lean mice (Fig. 7c).

TIMP2, which is the inhibitor of the late responding MMP2, shows a late response to the trauma and is increased in lean mice 192 h post-BTT (Fig. 7b). This response is decreased in obese animals leading to a statistically significant difference between the diets (Fig. 7d). Since the inhibition of MMP2 during the regeneration is an important factor, the lack of TIMP2 could again hint at a distorted response of obese mice to the trauma. Either obese animals are missing the response by organizing the ECM during tissue regeneration entirely, or these mice have not entered the state of tissue regeneration yet.

### Decreased total proteolytic MMP activity in lung tissue of HFD mice during ECM remodeling after BTT

**Fig. 8:**
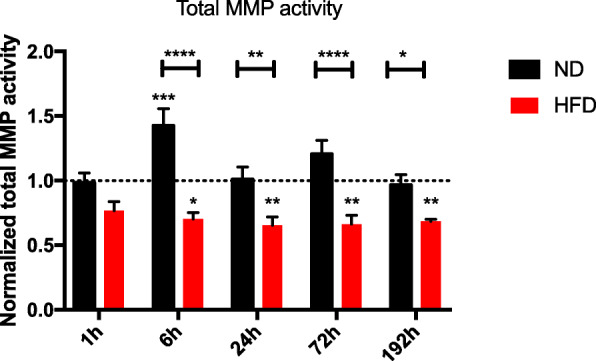
Analysis of total MMP activity in lung tissue of lean and obese animals after BTT. The total MMP activity was measured in the presence of naturally occurring inhibitors in lung tissue from control animals as well as 1 h, 6 h, 24 h, 72 h and 192 h post trauma. Statistical significance was determined using two-way ANOVA followed by an uncorrected Fisher’s LSD test (α = 0.05). * indicates p < 0.05, ** indicates p < 0.01, *** indicates p < 0.001, **** indicates p < 0.0001. Indicators of significance directly above the bars of the diagram indicate a statistical difference to the control, while stars above the connector line show differences between the two diets at that specific time point

An assay of total MMP activity was performed to evaluate the proteolytic potential of the pro-MMPs and MMPs in the presence of TIMPs naturally occurring in the analyzed lung tissue (Fig. 8). Lung tissue was homogenized and analyzed for this assay. The results show an increase of proteolytic MMP activity 6 h post trauma in lean mice and a constant decrease of MMP activity 24 h after trauma in obese animals. This leads to a statistically significant difference between the diets starting 6 h post BTT and indicating a distorted response in obese mice to the trauma. Since decreased MMP activity leads to a build-up of ECM, these results explain, why mice with the different diets result with the same amount of collagen 192 h after the trauma although the gene level indicates that lean mice have increased levels of ECM build-up genes compared to obese mice.

Collagen deposition is an important factor during repair of damaged tissue to ensure stability of the newly regenerated tissue if it gets removed after the regeneration phase. This can be achieved with active MMPs. Lean mice in contrast to obese mice seem to follow this regeneration principle of producing collagen followed by a digestion of the built ECM.

## Discussion

Obesity, which is characterized by dysfunctional adipose tissue and chronic inflammation, is a risk factor of acute respiratory distress syndrome (ARDS) even though it is still controversially discussed [36].

In this study, the effects of high-fat diet induced obesity on lung regeneration after blunt thorax trauma were investigated. The main objective was to elucidate whether morphologically changes and alterations on gene and protein levels during lung regeneration of HFD mice could relate to impaired regulation and organization of the ECM.

The investigation of ECM components included morphological assessment using SR staining, colorimetrical quantification of collagen content as well as gene expression analysis. The gene expression results were translated to the protein level using gelatin zymography as well as IHC staining. Morphological assessment utilizing Sirius Red staining showed locally increased amounts of collagen 192 h post-BTT in ND and HFD mice (Fig. 4a). Nevertheless, this increase was not detectable using colorimetrical quantification. This can be reasoned since the method behind the colorimetrical quantification investigates on a more comprehensive level (Fig. 4b). As lung injury was shown to increase lung weight, most likely to due to edema and the influx of interstitial cells, the quantification of collagen content 192 h post-BTT might be influenced when normalized to lung tissue weight [37–40].

However, comparable collagen deposition between the two diets was not expected since analysis of gene expression of *Col1a, Col3a* and *Fn1* showed an increase in their gene levels. This upregulation in later stages of regeneration was only observable in ND mice whereas HFD mice seemed to lack any response on gene level (Fig. 3). Nevertheless, building of ECM seems to be indispensable in lung regeneration after injury as it mainly contributes to structural stability and provides a scaffold involved in cell signaling and cell adhesion [41]. Thereby, collagen I is one of the major structural collagens and fibronectin is involved in cell adhesion of pulmonary cells [42]. As HFD mice did not show an upregulation in these crucial ECM genes post BTT, the response to the trauma might be hampered due to obesity which can result in an impact on lung regeneration process. A deficient upregulation of collagen in HFD mice has also been shown in a standard wound model [43] and additionally in an LPS-induced mild ARDS model [44]. Moreover, gene expression of procollagen I and III has been shown to be upregulated in ND mice but not in HFD mice after LPS-induced ALI [44], which is comparable to the results shown in this study.

Apart from a missing response in upregulation, post-translational processing of collagen and cross-linking of ECM could be disturbed in obesity. Since the outcome of collagen deposition is comparable between the diets, but gene level showed a stronger response in lean mice, a deregulation of ECM organization in HFD is highly likely. Therefore, gene expression levels of *Mmp2*, *Mmp8*, *Mmp9* and *Timp1* as well as *Timp2* were determined. *Mmp8*, *Mmp9* and *Timp1* were upregulated as a first response to injury (1 h to 6 h post-BTT) (Figs. 5 and 6) and *Mmp2* and its inhibitor *Timp2* were upregulated in the later progress of regeneration (72 h to 192 h post-BTT) (Figs. 5 and 6). The early responding genes *Mmp8* and *Mmp9* were statistically significantly higher expressed in ND mice compared to HFD animals. Additionally, the response of the respective inhibitor, *Timp1*, was delayed in obese mice. ND mice showed an upregulation of the gene at the same time the respective MMPs went up. However, this cannot be observed in obese mice, were the *Timp1* level was peaking 24 h post-BTT. The results of *Mmp9* translates to the protein level, proving MMP9 being an early responder to the trauma. Additionally, MMP9 activity was significantly decreased in HFD mice compared to lean mice post trauma. An involvement of MMP8 and MMP9 as early responders has been shown before in combination with a response due to pro-inflammatory cytokines TNF-α and interleukin 1 beta [45] induced gene expression of *Mmp9* in lung epithelial cells [46] as well as in eosinophils [47]. Apart from that, gene expression of *Mmp8* increased as a response to bleomycin-induced lung injury, most likely in leukocytes and fibroblasts [48]. Furthermore, MMP8 and MMP9 were both shown to be involved in the first inflammatory response after lung injury [49, 50].

During R phase an increase of *Mmp2* can be observed in lean mice. In other studies, MMP2 was shown to be involved in later stages of the regeneration process, especially in alveoli epithelial cell re-development after bleomycin-induced lung injury [51, 52]. Accordingly, controlled upregulation of MMPs after injury seems to be highly relevant to ensure a normal progress of regeneration. Considering the time-dependent gene expression of *Mmp2*, *Mmp8*, *Mmp9* and *Timp1* post-BTT, HFD mice failed to regulate the expression of these ECM-organizing components. This was further confirmed by increased MMP activity in lean mice starting from 6 h post BTT. This indicates ongoing remodeling processes as a normal part of the regeneration process. This increase in MMP activity is missing in obese mice. Since the activity even decreases after trauma it points to an uncontrolled build-up of ECM. Nevertheless, the exact mechanism behind this dysregulation of ECM remains unclear and needs to be further investigated. The missing regulation of ECM components as well as components that are necessary for organization of ECM, during the regeneration process in HFD mice could lead to an altered, pathological lung composition without sufficient stiffness and stability. Therefore, restricted lung function in HFD mice cannot be fully excluded.

## Conclusion

In this study, we screened lung regeneration and ECM regulation and organization at different time-points after blunt lung trauma in both, ND and HFD mice. HFD mice indicated more alveoli collapse, wall thickness as well as remaining filled alveoli structures in comparison to ND mice during the R phase after BTT. Although the gene levels of *Col1a*, *Col3a* and *Fn1* showed an upregulation in lean mice, it was missing in obese mice post trauma, Sirius Red staining and collagen counting did not show differences of collagen distribution between HFD mice and ND mice during the R phase. Therefore, the organization of the ECM was put into focus by performing gene expression analysis of several *Mmps* and *Timps* in addition to evaluation of the respective proteins. The results indicated that HFD mice were distinct in regulating ECM after lung injury and regeneration. Both, lean and obese mice, showed an initial response to the trauma by upregulating *Mmp8/9*. However, ND mice showed significant higher and longer increase of the gene levels which translates to the protein activity as well. The late responding *Mmp2* showed an increase in lean mice, which was missing in obese mice. The decreased gene level of all three investigated MMPs resulted in an overall decreased MMP activity in obese mice post-BTT. This might be the reason, that lean and obese mice show the same level of collagen deposition even though ND mice show increased collagen production on gene level compared to obese mice. Increased activity of MMPs in ND mice lead to a reconstruction of the deposited collagen that is necessary to restore proper lung tissue structure after injury. Although further investigation and verification is necessary, these results could hint at new strategies and new targets to influence the different healing process of obese trauma patients suffering from mild or severe ARDS. However, it needs to be mentioned that this study focused on male mice to reduce the number of animals needed for the experiments, due to the fact that previous studies have shown no difference between the two genders on this level of analysis. Since differences between males and females on obesity phenotypes have been described in humans before [53, 54], this is a limitation of the presented study regarding the translation to the human.

## Data Availability

The datasets used and/or analyzed during the current study are available from the corresponding author on reasonable request.

## Abbreviations

ARDS: Acute respiratory distress syndrome
BAL: Bronchoalveolar lavage
BTT: Blunt thorax trauma
BMI: Body mass index
CCL2: CC-chemokine-ligand-2
Col1a: Collagen 1a
Col3a: Collagen 3a
DI phase: Damage and inflammation phase
ECM: Extracellular matrix
G-CSF: Granulocyte colony stimulating factor
Fn1: Fibronectin
HFD: High fat diet
HE: Hematoxylin and eosin
IVC: Individually ventilated cage
LPS: Lipopolysaccharide
MMPs: Matrix metallopeptidases
ND: Normal diet
qPCR: quantitative polymerase chain reaction
R phase: Repair phase
rbcs: red blood cells
RT: Room temperature
SR: Sirius red
TIMPs: Tissue inhibitors of metalloproteinases
TNF-α: Tumor necrosis factor alpha
